# Assessing the Need and Acceptance of a Smart Asthma Management Application Among Patients in Saudi Arabia

**DOI:** 10.3390/healthcare14203497

**Published:** 2026-10-17

**Authors:** Manjur Kolhar, Yousef Saad Aldabayan

**Affiliations:** 1 Department of Health Information Management and Technology, College of Applied Medical Sciences, King Faisal University, Al-Ahsa 31982, Saudi Arabia; 2 Department of Respiratory Care, College of Applied Medical Sciences, King Faisal University, Al-Ahsa 31982, Saudi Arabia; yaldabayan@kfu.edu.sa

**Keywords:** asthma, mobile health, digital health, self-management, mobile applications, environmental exposure, Saudi Arabia

## Abstract

Background: Digital health technologies can support asthma self-management through symptom tracking, medication reminders, environmental alerts, and communication with healthcare providers. However, evidence regarding patients’ perceived needs and acceptance of an integrated smart asthma management application in Saudi Arabia remains limited. Objective: This study aimed to assess perceived need for and intended acceptance of a Saudi-based smart asthma management application among adults with asthma and identify factors associated with their likelihood of using the proposed application. Methods: A cross-sectional questionnaire-based study was conducted among adults with asthma in Saudi Arabia. The questionnaire assessed asthma self-management experiences, environmental challenges, previous digital health application use, and preferences for proposed application features. Descriptive statistics, Spearman’s rank correlations, and ordinal logistic regression were performed. Internal consistency of the five core feature-needs items was evaluated using Cronbach’s alpha. The Benjamini–Hochberg false discovery rate procedure was applied to adjust for multiple correlation comparisons. Results: A total of 68 adults were included in the final analysis. Overall, 77.9% reported a moderate-to-high need for the proposed application, 58.8% expressed moderate-to-high willingness to use it, and 66.2% indicated that they were likely or very likely to use a Saudi-based smart asthma application. The five core feature-needs items demonstrated high internal consistency (Cronbach’s α = 0.899). Overall perceived need showed the strongest positive correlation with likelihood of use (ρ = 0.709, 95% CI: 0.545–0.844, FDR-adjusted *q* < 0.001). Significant positive correlations were also observed for environmental alerts (ρ = 0.683), Sehhaty integration (ρ = 0.674), core feature-needs score (ρ = 0.667), perceived environmental burden (ρ = 0.646), and smartwatch connectivity (ρ = 0.590), all *q* < 0.001. In the adjusted ordinal logistic regression model, perceived environmental burden (aOR = 4.09, 95% CI: 2.09–7.98, *p* < 0.001) and need for additional asthma-management support (aOR = 3.17, 95% CI: 1.62–6.21, *p* < 0.001) were independently associated with greater likelihood of intended application use. Conclusions: Adults with asthma demonstrated substantial perceived need for and willingness to use a Saudi-based smart asthma management application. Perceived environmental burden and need for additional asthma-management support were particularly associated with intended use. These findings support user-centered development and prospective evaluation of an integrated application incorporating contextually relevant asthma self-management and digital health features.

## 1. Introduction

Asthma is a chronic respiratory disease characterized by variable respiratory symptoms and airflow limitation and requires continuous management to achieve symptom control and reduce the risk of exacerbations [1,2,3]. Effective asthma care extends beyond pharmacological treatment and includes patient education, adherence to prescribed therapy, recognition and avoidance of triggers, symptom monitoring, appropriate response to worsening symptoms, and communication with healthcare professionals [4,5,6]. Because many of these activities occur outside healthcare facilities, effective self-management is an important component of long-term asthma care [7,8]. Nevertheless, maintaining appropriate self-management in daily life can be challenging, particularly when patients must simultaneously manage medications, symptoms, environmental exposures, appointments, and individualized treatment recommendations [9,10].

Digital health technologies have created new opportunities to support asthma self-management outside conventional clinical settings [11,12]. Smartphone applications, web-based platforms, wearable devices, electronic medication-monitoring systems, and remote communication technologies can facilitate symptom monitoring, medication reminders, patient education, personalized feedback, and communication between patients and healthcare professionals [13,14]. The findings indicate that digital interventions may complement conventional asthma care, while also emphasizing that their effectiveness depends on intervention design, functionality, patient engagement, and clinical context.

An important consideration in developing digital asthma interventions is whether their functions correspond to the actual needs and preferences of intended users [15,16,17,18,19]. Asthma self-management applications have incorporated symptom tracking, medication reminders, education, clinical support, action plans, and communication functions [20,21,22]. More recent digital interventions have also incorporated wearable-device integration and tailored notifications [23,24,25,26]. However, technological complexity does not necessarily translate into patient acceptance or sustained engagement [27,28,29]. User-centered development is therefore important to identify which functions patients consider relevant and how digital tools can be integrated into their existing self-management practices and healthcare environment. The NASSS (Non-adoption, abandonment, scale-up, spread, and sustainability) tool, too, considers the intended users of technology, and the practices they already employ. The interactions between technology and other contexts can adapt and evolve. A user-centered design approach therefore can be supported by identifying functions that patients believe to be relevant, and then designing the application to support patients’ current management practices via technology.

This consideration is particularly relevant in Saudi Arabia, where the expanding digital-health infrastructure provides opportunities for mobile-supported chronic disease management. Previous research among adults with asthma in Saudi Arabia has demonstrated considerable interest in using mobile technologies for asthma management, despite relatively limited use of asthma-specific applications [30,31,32,33,34]. This indicates a need to better understand patients’ expectations, preferences, and perceived needs before developing context-specific digital asthma interventions.

Healthcare professionals in Saudi Arabia have also identified functions that could be relevant to the development of asthma applications. A survey of Saudi healthcare providers reported strong support for functions including medication reminders, information regarding asthma triggers, recording asthma attacks and medication use, mechanisms for requesting assistance during exacerbations, and connectivity between patients, healthcare providers, and healthcare centers [30,34,35,36]. These findings provide an important professional perspective on application design; however, patient-centered assessment remains necessary because healthcare-provider priorities may not fully represent the needs, preferences, and intended use of individuals living with asthma.

The Saudi digital-health ecosystem provides additional opportunities for developing integrated rather than isolated asthma-management tools [37,38,39,40]. Sehhaty, the unified digital-health platform of the Saudi Ministry of Health, provides access to services including appointments, remote consultations, medications and medical reports, health information, vital-sign monitoring, and educational resources [41,42]. Importantly, the platform also provides early warnings of weather conditions for people with asthma. The presence of an established national platform raises questions regarding whether future asthma-management interventions should interact with existing digital-health infrastructure and whether users value such interoperability. Wearable technologies, including smartwatches, may provide further opportunities to integrate patient-generated health information with mobile self-management systems.

Environmental exposure is another potentially important dimension of digitally supported asthma management in Saudi Arabia. Air pollution, particulate matter, dust, and changing weather conditions may influence respiratory symptoms and asthma exacerbations. Assessing patient perceptions of environmental burden and interest in environmental monitoring is therefore relevant when considering the design of context-specific asthma technologies.

Previous Saudi studies have examined patients’ use of mobile phones for asthma care and healthcare professionals’ preferences for asthma-application features, but evidence remains limited regarding patients’ perceived need for an integrated smart asthma management application. In particular, further evidence is needed regarding preferences for environmental monitoring, symptom tracking, medication support, emergency functions, information sharing with healthcare providers, integration with national digital-health services, and wearable connectivity. It is also important to understand how asthma-management experiences, environmental factors, and previous digital-health use are associated with intended adoption of such technology.

This study explores the perceived need and acceptance of a smart asthma management application by individuals with asthma in Saudi Arabia. The study examines their current asthma self-management practices, environmental burden, use of existing digital health applications, desired features of a smart asthma management application and their willingness to use a Saudi-developed smart asthma management application. In addition, the study investigates factors that could influence the intended use of the proposed application. The findings from this study will aid in the user-centered design and evaluation of a smart asthma management intervention that is context specific to Saudi Arabia.

## 2. Materials and Methods

### 2.1. Study Design and Setting

A cross-sectional study was conducted in Saudi Arabia to explore the perceived need for and willingness to use a smart asthma management application among adults with asthma. Participants completed a structured questionnaire assessing demographic and asthma-related characteristics, asthma self-management, perceived environmental factors affecting asthma, use of existing digital health applications, preferred features of a proposed application, and likelihood of using a Saudi-based smart asthma management application.

### 2.2. Participants and Recruitment

Adults aged 18 years or older with asthma and residing in Saudi Arabia were included in the present analysis. Participation was voluntary, and informed consent was required before completion of the questionnaire. Responses from participants younger than 18 years and those who did not provide informed consent were excluded from the final analytical sample. Accordingly, the final analysis included 68 adult participants. Pediatric asthma self-management was outside the scope of the present analysis.

### 2.3. Survey Instrument

Data was collected using a structured, self-administered electronic questionnaire developed specifically for this study to assess asthma-management experiences, digital-health use, environmental factors, and perceived needs related to a proposed smart asthma management application. The questionnaire was available in both Arabic and English and administered through Google Forms. The survey was distributed through social media platforms between 17 April and 30 August 2026 using a nonprobability convenience sampling approach. Although the internal consistency of the five core feature-needs items was assessed using Cronbach’s alpha, the complete questionnaire did not undergo formal psychometric validation. The questionnaire included items covering demographic and asthma-related characteristics, symptom frequency and asthma control, healthcare utilization, medication adherence, self-management experiences, access to asthma-related information, previous use of digital-health applications, environmental factors affecting asthma, preferences for proposed application features, and intended application use. The proposed features included environmental alerts, medication reminders, symptom tracking, physician-report sharing, emergency alerts, integration with the Saudi national health application Sehhaty, and smartwatch connectivity. Participants were also asked about their overall perceived need for the proposed application, willingness to use it, and likelihood of using a Saudi-based smart asthma management application.

Feature-need items were assessed using four-category ordinal response scales ranging from *not needed* to *highly needed*. Five feature-related items—environmental alerts, medication reminders, symptom tracking, physician-report sharing, and emergency alerts—were combined to form a core feature-needs score, with higher scores indicating greater perceived need for smart asthma-management functions. Internal consistency of these five items was high in the final adult sample (Cronbach’s α = 0.899).

Environmental burden was assessed using two items examining the perceived effects of dust storms and air pollution on participants’ asthma. Both items were assessed using four-category ordinal response scales, with higher scores indicating greater perceived environmental impact. The two items were combined to derive the environmental burden score used in the correlation and regression analyses.

Acceptance-related outcomes were assessed separately through participants’ overall perceived need for a smart asthma application, willingness to use the application, and likelihood of using a Saudi-based smart asthma management application. These variables were analyzed as individual ordinal measures rather than as components of a single validated acceptance scale.

### 2.4. Study Variables and Scoring

The primary outcome for the association analyses was participants’ likelihood of using a Saudi-based smart asthma management application. Responses were recorded on a four-category ordinal scale ranging from 1 (very unlikely) to 4 (very likely).

A core feature-needs score was derived from five proposed application features: environmental alerts, medication reminders, symptom tracking, physician-report sharing, and emergency alerts. Each feature was rated on a four-point scale ranging from 1 (not needed) to 4 (highly needed), with higher scores indicating greater perceived need. A composite score was calculated from these items for each participant.

Environmental burden was assessed using two questionnaire items measuring the perceived effects of dust storms and air pollution on participants’ asthma. Each item was coded on a four-point scale: 1 (no effect), 2 (low effect), 3 (moderate effect), and 4 (high effect). The environmental burden score was calculated as the mean of the two item scores, resulting in a possible range of 1–4, with higher scores indicating greater perceived environmental burden. A one-point increase therefore represents a one-point increase on this composite perceived environmental-burden scale and should not be interpreted as a clinically validated change in environmental exposure.

Other variables considered in the association analyses included perceived difficulty managing asthma without digital support, need for additional asthma-management support, previous use of general health applications, and preferences for specific digital health features.

### 2.5. Data Collection

The questionnaire was administered electronically. Participants received information about the purpose of the study and provided informed consent before proceeding to the survey questions. Responses were collected anonymously. Data were reviewed before analysis, and responses from participants younger than 18 years and those who did not provide informed consent were excluded from the final analytical dataset.

### 2.6. Statistical Analysis

Descriptive statistics were used to summarize participant characteristics and questionnaire responses. Categorical variables were reported as frequencies and percentages, while composite and ordinal measures were summarized using appropriate descriptive statistics. Statistical analyses were performed using Python (version 3.12.7, Python Software Foundation, Wilmington, DE, USA) with the pandas, NumPy, SciPy, and statsmodels libraries.

Of the 76 completed questionnaires, eight responses from participants younger than 18 years were excluded, resulting in a final analytical sample of 68 adults. Complete observations were available for all variables included in the correlation and ordinal logistic regression analyses; therefore, no imputation was required.

The internal consistency of the five core feature-needs items was assessed using Cronbach’s alpha. Spearman’s rank correlation coefficient (ρ) was used to examine associations between selected participant, asthma-management, environmental, and digital-health factors and the likelihood of using the proposed application. Spearman correlation was selected because the primary outcome and several explanatory variables were ordinal or derived from ordinal questionnaire responses. Ninety-five percent confidence intervals (CIs) for Spearman’s correlation coefficients were estimated using percentile bootstrap resampling with 3000 iterations. To account for multiple comparisons, the Benjamini–Hochberg false discovery rate (FDR) procedure was applied across the 23 correlation tests. Both unadjusted *p*-values and FDR-adjusted q-values were reported, with *q* < 0.05 considered statistically significant.

An ordinal logistic regression model was used to examine factors independently associated with the likelihood of using the proposed application. The dependent variable was likelihood of use, coded from 1 (very unlikely) to 4 (very likely). The model included difficulty managing asthma without digital support, previous use of general health applications, perceived environmental burden, and perceived need for additional asthma-management support as predictors. Regression coefficients (β), standard errors (SE), adjusted odds ratios (aORs), 95% CIs, and *p*-values were reported. An aOR greater than 1 indicated greater odds of being in a higher likelihood-of-use category, conditional on the other predictors. Regression analyses were considered exploratory, and statistical significance was assessed using two-sided *p*-values < 0.05.

Given the cross-sectional design and self-reported nature of the data, the findings were interpreted as associations rather than evidence of causality or actual application adoption.

### 2.7. Ethical Considerations

Participation was voluntary, and informed consent was obtained before respondents proceeded with the questionnaire. Only participants aged 18 years or older were included in the final analysis. Participant information was handled in accordance with the approved study procedures and applicable ethical requirements. Ethical approval was obtained from the Research Ethics Committee at King Faisal University, Saudi Arabia (KFU-REC-2026-APR-ETHICS4146; approved on 16 April 2026).

## 3. Results

### 3.1. Demographic and Clinical Characteristics

After excluding participants younger than 18 years, a total of 68 adult participants with asthma were included in the final analysis. The largest age group was 18–30 years (*n* = 30, 44.1%), followed by those aged 31–45 years (*n* = 18, 26.5%), 46–60 years (*n* = 15, 22.1%), and older than 60 years (*n* = 5, 7.4%). Most participants were female (*n* = 49, 72.1%) and resided in the Eastern Region of Saudi Arabia (*n* = 64, 94.1%). Nearly two-thirds held a bachelor’s degree (*n* = 43, 63.2%).

The duration since asthma diagnosis varied among participants. Twenty-three participants (33.8%) had been diagnosed with asthma for more than 10 years, 20 (29.4%) for 1–5 years, 13 (19.1%) for 6–10 years, and 12 (17.6%) for less than 1 year. Asthma symptoms occurred sometimes in 25 participants (36.8%), rarely in 22 (32.4%), often in 16 (23.5%), and always in 5 (7.4%).

Regarding asthma control, 30 participants (44.1%) rated their asthma control as fair, 17 (25.0%) as excellent, 16 (23.5%) as good, and 5 (7.4%) as poor. Healthcare utilization also varied. Twenty-four participants (35.3%) reported no previous emergency department visits due to asthma, whereas 44 (64.7%) reported at least one emergency department visit. Asthma-related hospitalization was less common, with 48 participants (70.6%) reporting no previous hospitalization and 20 (29.4%) reporting at least one hospitalization.

The impact of asthma on daily life was rated as low by 35 participants (51.5%), moderate by 22 (32.4%), and high by 11 (16.2%). Medication adherence also varied, with 32 participants (47.1%) reporting that they sometimes adhered to their asthma medication, 16 (23.5%) reporting never, 14 (20.6%) reporting often, and 6 (8.8%) reporting always (Table 1).

### 3.2. Asthma Self-Management and Digital Health Experience

The levels of difficulties the participants faced, as well as their level of confidence in managing their asthma, were also assessed (see Table 2). Almost half of the participants (48.7%, *n* = 37) did not face any problems managing their asthma without the use of digital support. A slight problem was encountered by 36.8% of the participants (*n* = 28), followed by 10.5% (*n* = 8) of the participants experiencing moderate problems. In total, 51.3% of the participants (*n* = 39) did have some kind of problem managing their asthma without the use of digital support. Despite these differences, participants generally reported moderate to high levels of confidence in managing their asthma. Moderate confidence was reported by 30 participants (39.5%), while 17 (22.4%) reported high confidence and 18 (23.7%) reported very high confidence. Eleven participants (14.5%) reported low confidence. Thus, 65 participants (85.5%) reported at least moderate confidence in their ability to self-manage asthma.

Access to asthma-related education and information was less consistent. Twenty-nine participants (38.2%) reported having sufficient access to asthma education and information, while 34 (44.7%) indicated that they had sufficient access only sometimes and 13 (17.1%) reported insufficient access. Regarding additional support for asthma management, 34 participants (44.7%) reported a slight need, 9 (11.8%) a moderate need, and 13 (17.1%) a high need. Overall, 56 participants (73.7%) expressed at least some need for additional asthma-management support.

Regarding digital health experience, slightly more than half of the participants had previously used a health-related mobile application (*n* = 36, 52.9%). However, experience with asthma-specific applications was very limited, with only 3 participants (4.4%) reporting previous use of an asthma-management application. Most participants reported a neutral level of satisfaction with health applications (*n* = 50, 73.5%), while 11 (16.2%) were very satisfied, 5 (7.4%) were dissatisfied, and 2 (2.9%) were very dissatisfied (Table 2).

### 3.3. Environmental Factors and Environmental Support Needs

Environmental factors appeared to be relevant to asthma symptoms among the participants. As shown in Table 3, dust storms were reported to have a high effect on asthma symptoms by 31 participants (45.6%) and a moderate effect by 16 (23.5%). Overall, 47 participants (69.1%) reported a moderate or high impact of dust storms on their asthma symptoms. Fourteen participants (20.6%) reported a low effect, while 7 (10.3%) reported no effect.

Air pollution showed a similar pattern. Thirty-six participants (52.9%) reported a high effect of air pollution on their asthma symptoms, while 15 (22.1%) reported a moderate effect. Collectively, 51 participants (75.0%) reported a moderate or high effect of air pollution. Twelve participants (17.6%) reported a low effect, while 5 (7.4%) reported no effect (Table 3).

Air-quality monitoring practices varied among participants. Fifteen participants (22.1%) reported that they never monitored air quality, while 28 (41.2%) did so sometimes, 15 (22.1%) often, and 10 (14.7%) always. Participants also showed considerable interest in receiving environmental information through the proposed application. Environmental alerts were rated as highly needed by 32 participants (47.1%) and moderately needed by 16 (23.5%). Together, 48 participants (70.6%) expressed a moderate-to-high need for this feature. In comparison, 11 (16.2%) reported a slight need, while 9 (13.2%) indicated no need for environmental alerts (Table 3).

### 3.4. Preferred Smart Asthma Application Features

**Figure 1:**
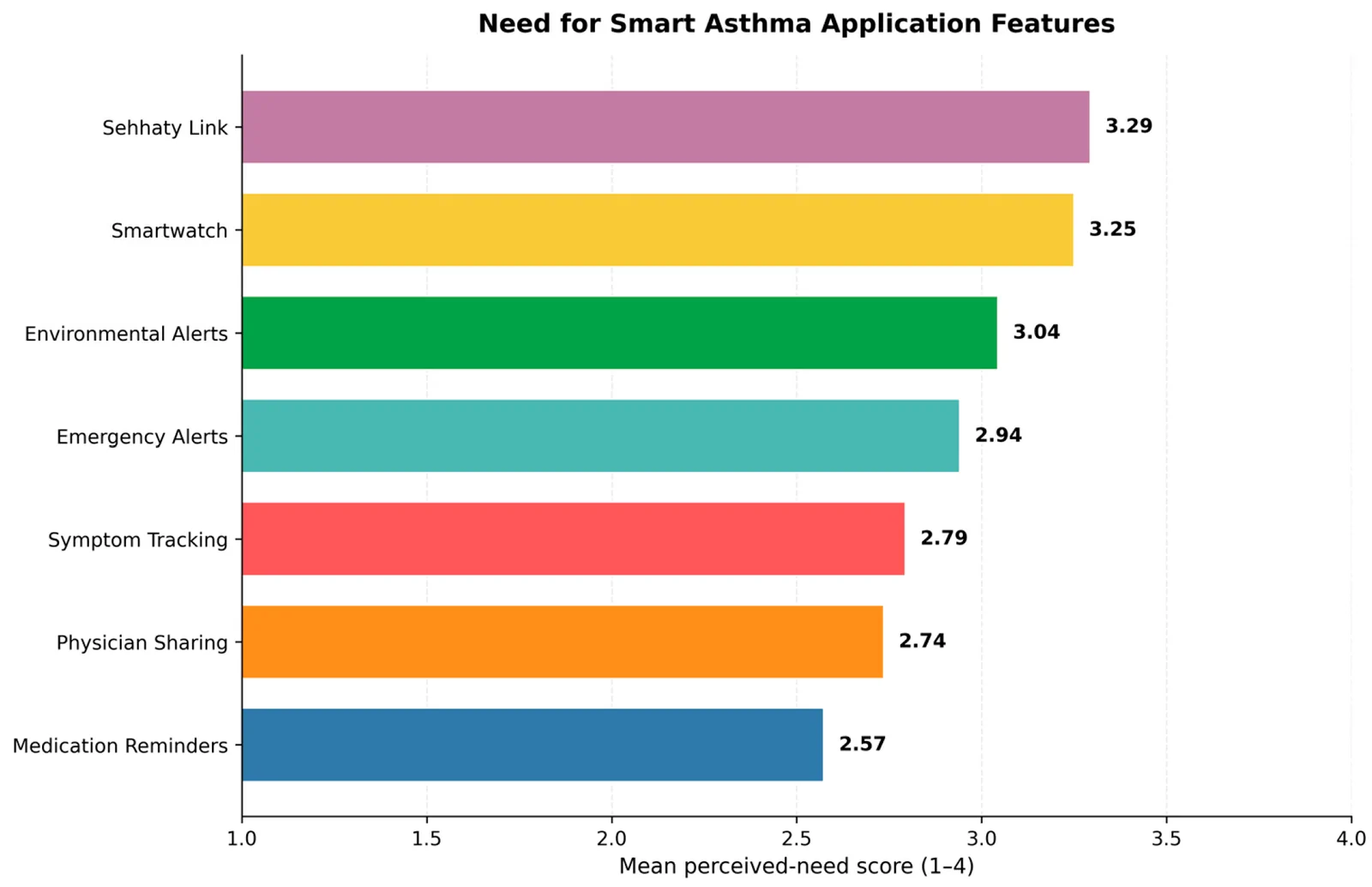
Mean perceived-need scores for proposed smart asthma application features among adult participants (*n* = 68). Scores ranged from 1 (not needed) to 4 (highly needed).

Participants showed considerable interest in a range of features proposed for the smart asthma management application. As presented in Table 4 and Figure 1, integration with Sehhaty received the highest mean perceived-need score (mean = 3.29, SD = 0.95), followed by smartwatch connectivity (mean = 3.25, SD = 1.01) and environmental alerts (mean = 3.04, SD = 1.08). Emergency alerts also received a relatively high mean score (mean = 2.94, SD = 1.17), followed by symptom tracking (mean = 2.79, SD = 1.10), physician-report sharing (mean = 2.74, SD = 1.14), and medication reminders (mean = 2.57, SD = 1.25).

When individual response categories were considered, Sehhaty integration and smartwatch connectivity were each rated as highly needed by 39 participants (57.4%). Environmental alerts and emergency alerts were each rated as highly needed by 32 participants (47.1%). Symptom tracking and physician-report sharing were each rated as highly needed by 24 participants (35.3%), while medication reminders were rated as highly needed by 23 participants (33.8%).

When moderate and high need categories were combined, 53 participants (77.9%) expressed a moderate-to-high need for Sehhaty integration, followed by smartwatch connectivity (*n* = 52, 76.5%), environmental alerts (*n* = 48, 70.6%), emergency alerts (*n* = 44, 64.7%), symptom tracking (*n* = 41, 60.3%), physician-report sharing (*n* = 39, 57.4%), and medication reminders (*n* = 37, 54.4%). These findings indicate interest in multiple application functions, particularly integration with existing digital health services, wearable connectivity, environmental monitoring, and emergency-support capabilities (Table 4; Figure 1).

The five core application-feature items—environmental alerts, medication reminders, symptom tracking, physician-report sharing, and emergency alerts—demonstrated excellent internal consistency (Cronbach’s α = 0.899). These five items were combined to form an overall core feature-need score for subsequent analyses.

### 3.5. Overall Need and Acceptance of the Smart Asthma Application

**Figure 2:**
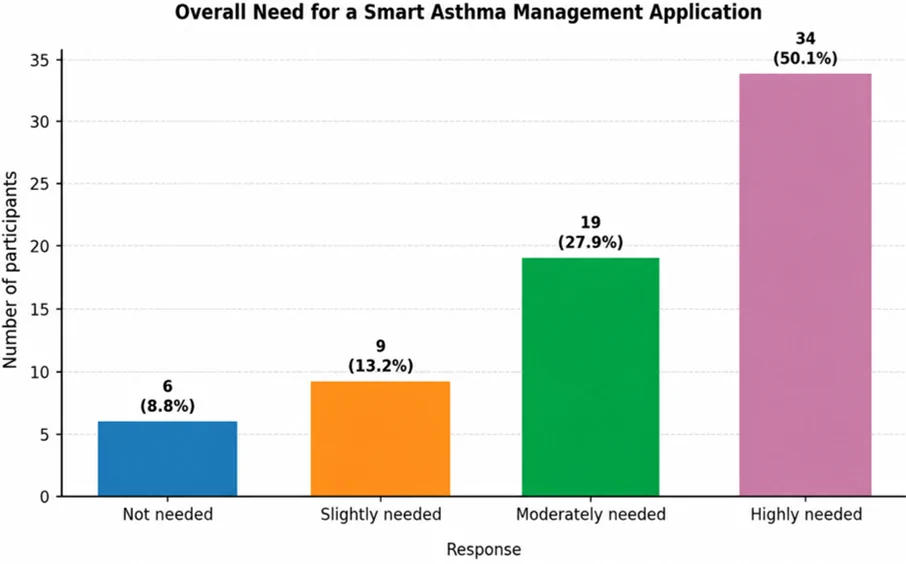
Overall perceived need for a smart asthma management application among participants (*n* = 68).

Participants generally expressed a favorable perceived need for the proposed smart asthma management application. As shown in Table 5 and Figure 2, 34 participants (50.0%) rated the application as highly needed, while 19 (27.9%) considered it moderately needed. Nine participants (13.2%) considered it slightly needed, and 6 (8.8%) considered it not needed. Overall, 53 participants (77.9%) considered the application moderately or highly needed.

**Figure 3:**
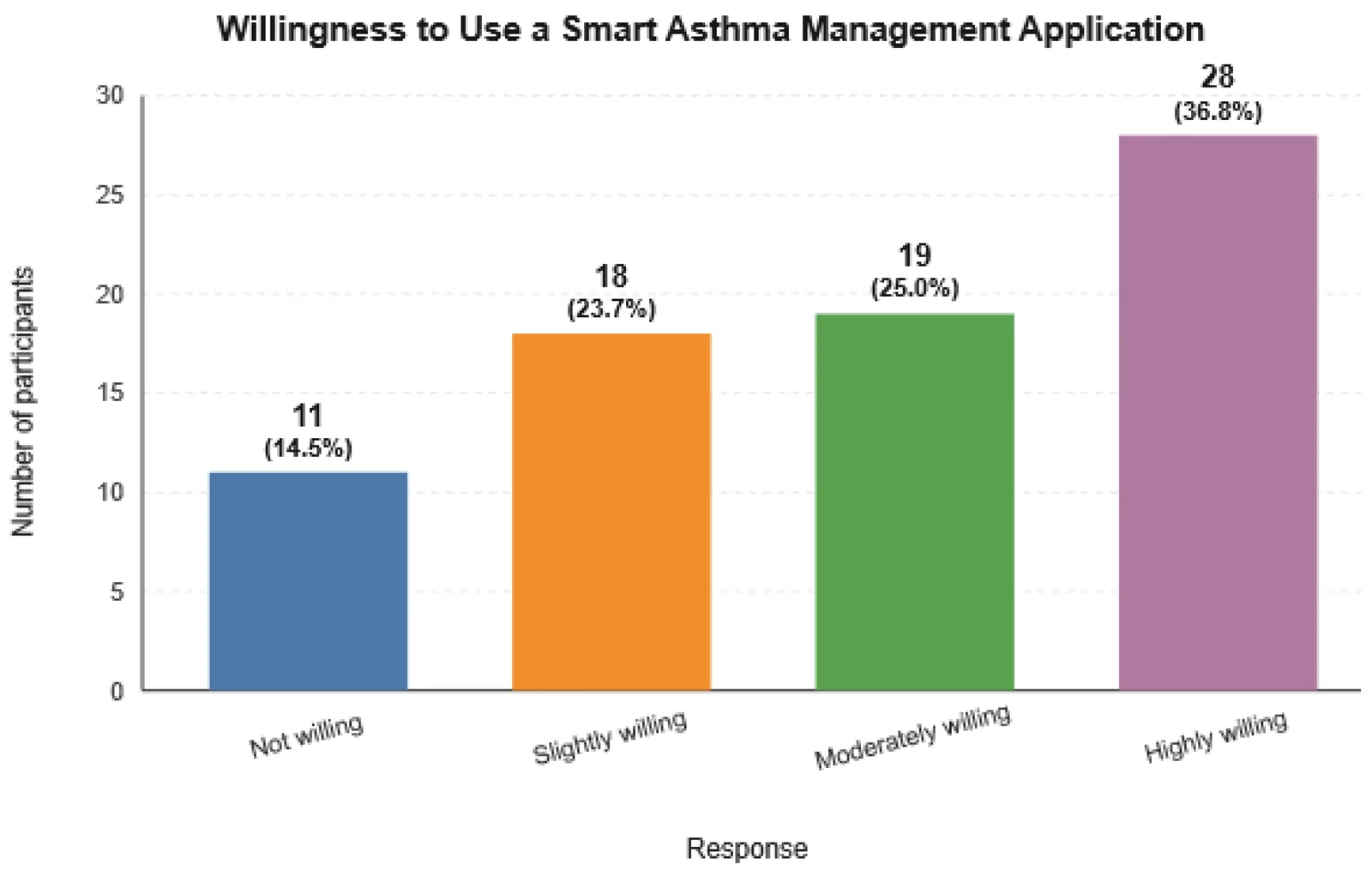
Participants’ willingness to use a smart asthma management application (*n* = 68).

Regarding willingness to use the application, 23 participants (33.8%) were highly willing and 17 (25.0%) were moderately willing. Eighteen participants (26.5%) were slightly willing, while 10 (14.7%) were not willing. Thus, 40 participants (58.8%) were moderately or highly willing to use a smart asthma management application (Table 5; Figure 3).

**Figure 4:**
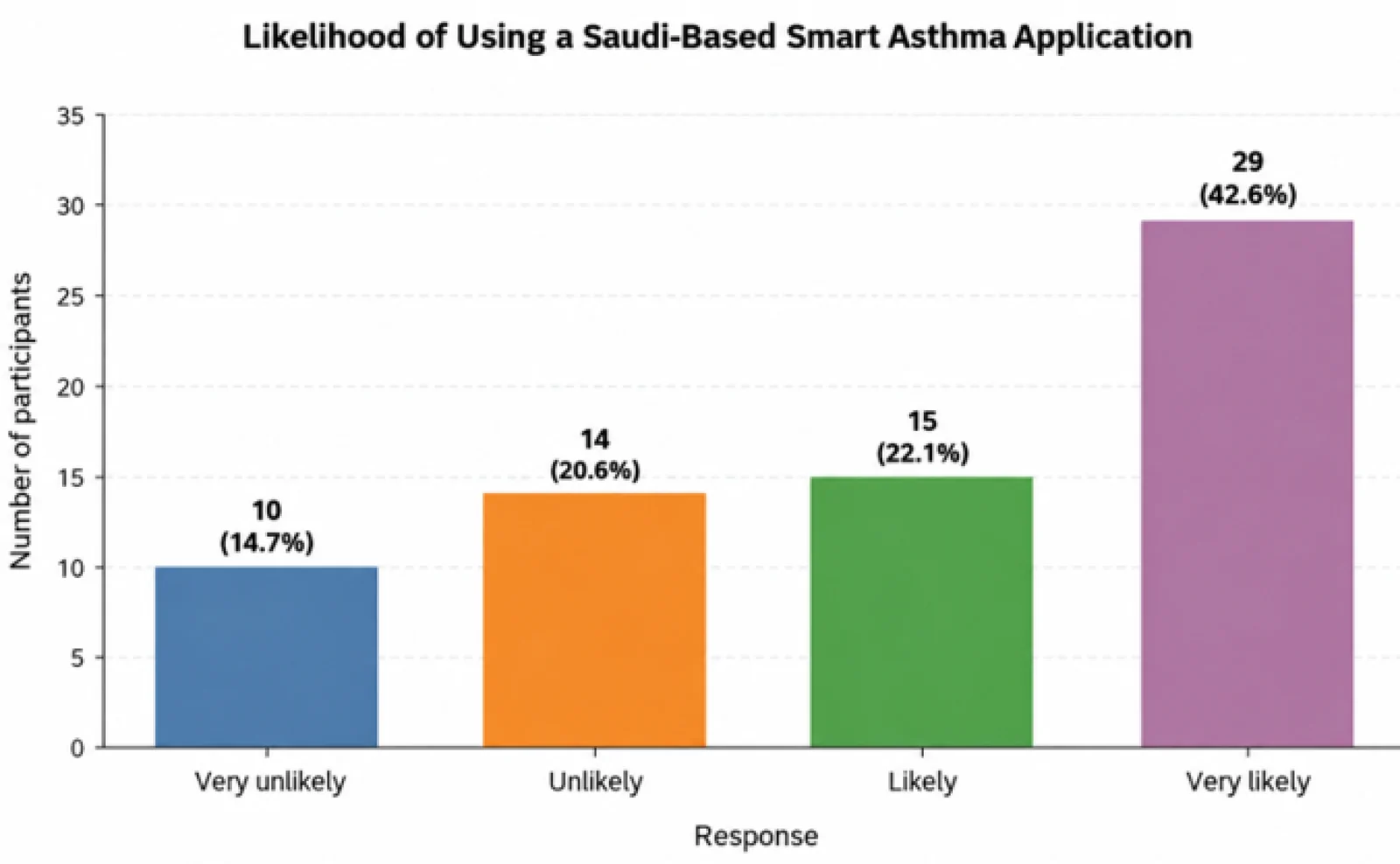
Likelihood of using a Saudi-based smart asthma management application among participants (*n* = 68).

Similarly, intention to use a Saudi-based smart asthma application was favorable. Twenty-nine participants (42.6%) reported being very likely to use such an application, while 16 (23.5%) were likely to use it. Twelve participants (17.6%) were unlikely, and 11 (16.2%) were very unlikely. Overall, 45 participants (66.2%) were likely or very likely to use a Saudi-based smart asthma application (Table 5; Figure 4).

Overall, the findings showed a generally favorable pattern of perceived need and acceptance. More than three-quarters of participants (77.9%) reported a moderate-to-high need for the application, 58.8% were moderately or highly willing to use it, and 66.2% indicated that they were likely or very likely to use a Saudi-based smart asthma application.

**Figure 5:**
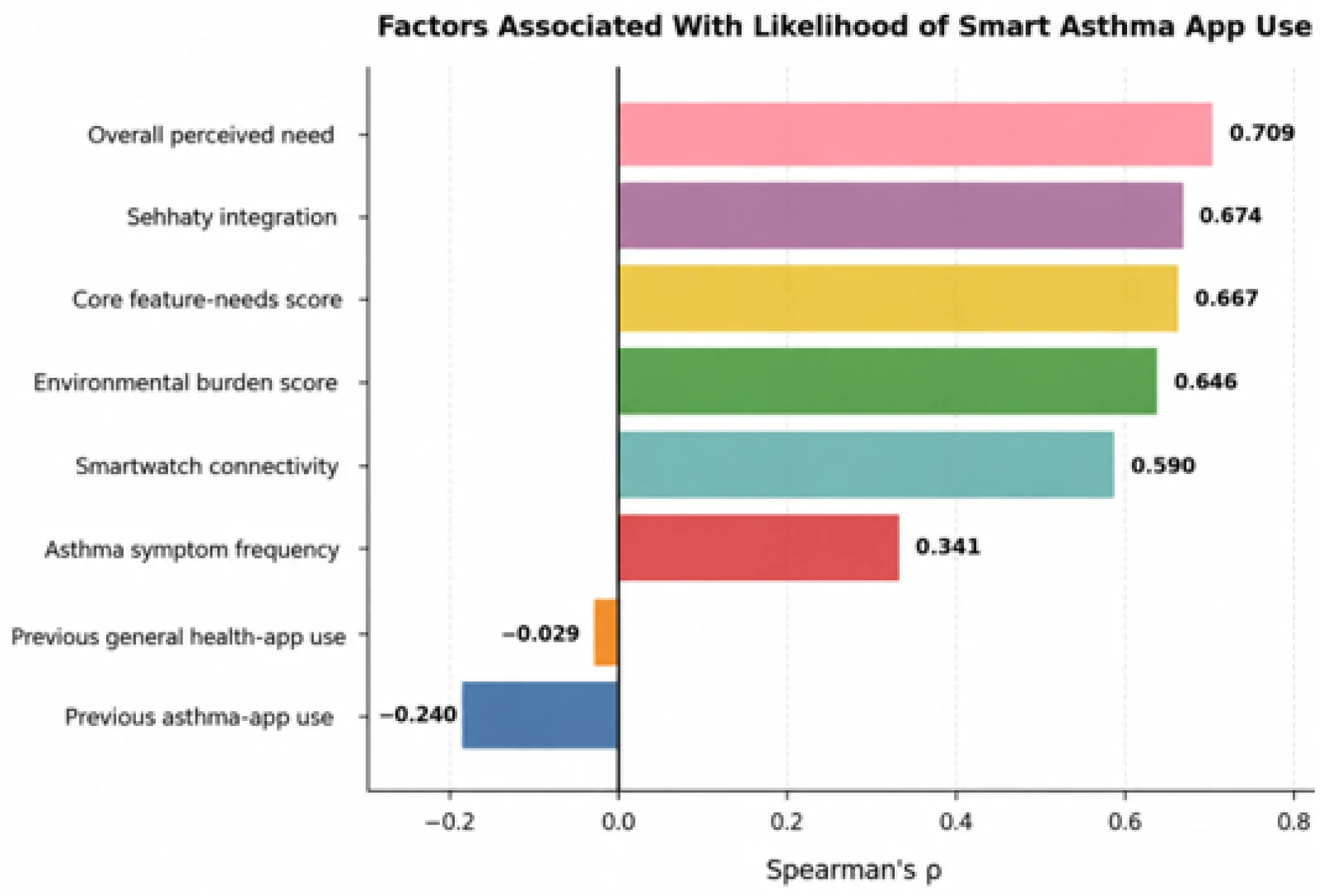
Factors associated with the likelihood of using a Saudi-based smart asthma management application among participants (*n* = 68).

Spearman’s rank correlation analysis was conducted to examine factors associated with participants’ likelihood of using a Saudi-based smart asthma management application. As presented in Table 6 and Figure 5, 17 of the 23 examined factors remained statistically significant after adjustment for multiple comparisons using the Benjamini–Hochberg false discovery rate (FDR) procedure (*q* < 0.05).

The strongest positive association was observed between overall perceived need and likelihood of application use (ρ = 0.709, 95% CI: 0.545–0.844, *q* < 0.001). Other strong positive associations were identified for environmental alerts (ρ = 0.683), Sehhaty integration (ρ = 0.674), the core feature-needs score (ρ = 0.667), emergency alerts (ρ = 0.655), perceived environmental burden (ρ = 0.646), symptom tracking (ρ = 0.632), willingness to use the application (ρ = 0.622), dust-storm impact (ρ = 0.615), and smartwatch connectivity (ρ = 0.590), all with FDR-adjusted *q*-values < 0.001.

Additional statistically significant positive associations were observed for air-pollution impact (ρ = 0.580), need for additional asthma-management support (ρ = 0.565), and physician-report sharing (ρ = 0.508), all *q* < 0.001. Moderate-to-weak positive associations were identified for the daily-life impact of asthma (ρ = 0.402, *q* = 0.001), medication reminders (ρ = 0.385, *q* = 0.002), difficulty managing asthma without digital support (ρ = 0.373, *q* = 0.003), and asthma symptom frequency (ρ = 0.341, *q* = 0.006).

No statistically significant associations were observed after FDR adjustment for air-quality monitoring (ρ = 0.209, *q* = 0.100), access to asthma education or information (ρ = 0.097, *q* = 0.471), self-management confidence (ρ = −0.021, *q* = 0.868), previous general health-app use (ρ = −0.029, *q* = 0.850), or asthma control (ρ = −0.229, *q* = 0.073). Previous asthma-app use demonstrated a weak negative correlation with intended application use (ρ = −0.240, unadjusted *p* = 0.048); however, this association did not remain statistically significant after FDR adjustment (*q* = 0.062). This finding also warrants caution because only three participants reported previous asthma-app use.

Overall, intended application use was most strongly associated with perceived need, preferences for digital features and healthcare-system integration, and perceived environmental burden. These exploratory findings highlight factors potentially relevant to application design and future implementation research but do not establish causal relationships or predict actual application adoption.

As shown in Table 6 and Figure 5, likelihood of using a Saudi-based smart asthma management application was positively associated with several perceived-need, feature-preference, environmental, and disease-management factors. The strongest association was observed for overall perceived need (ρ = 0.709, *q* < 0.001), followed by environmental alerts (ρ = 0.683, *q* < 0.001), Sehhaty integration (ρ = 0.674, *q* < 0.001), the core feature-needs score (ρ = 0.667, *q* < 0.001), emergency alerts (ρ = 0.655, *q* < 0.001), and perceived environmental burden (ρ = 0.646, *q* < 0.001). Willingness to use the application was also positively associated with likelihood of use (ρ = 0.622, *q* < 0.001). Overall, 17 of the 23 examined factors remained statistically significant after Benjamini–Hochberg false discovery rate (FDR) adjustment. In contrast, air-quality monitoring, asthma control, access to asthma education/information, self-management confidence, and previous general health-app use were not significantly associated with likelihood of use after adjustment (all *q* > 0.05). Previous asthma-app use showed a weak negative association (ρ = −0.240, unadjusted *p* = 0.048), but this association did not remain significant after FDR adjustment (*q* = 0.062). This finding should be interpreted cautiously because only three participants reported previous asthma-app use.

### 3.6. Predictors of Likelihood of Using the Smart Asthma Application

An ordinal logistic regression analysis was conducted to examine factors independently associated with participants’ likelihood of using a Saudi-based smart asthma management application. The model included management difficulty, previous use of general health applications, environmental burden, and need for additional asthma-management support as predictors. Complete data were available for the outcome and all four predictors among the 68 adult participants, so no imputation was required.

Model diagnostics indicated no evidence of problematic multicollinearity, with variance inflation factors ranging from 1.007 to 1.402 and tolerance values ranging from 0.713 to 0.993. Examination of predictor coefficients across the three cumulative outcome thresholds showed consistent coefficient directions and particularly stable estimates for environmental burden, providing reasonable support for the proportional-odds specification, although some variation in coefficient magnitude was observed for the other predictors. The full model provided a significantly better fit than the null model (likelihood-ratio χ^2^(4) = 51.687, *p* < 0.001). The fitted model had a log-likelihood of −62.875, AIC of 139.749, and BIC of 155.286.

As shown in Table 7, perceived environmental burden was significantly associated with greater likelihood of application use (aOR = 4.09, 95% CI: 2.09–7.98, *p* < 0.001). For each one-point increase in the mean perceived environmental burden score (range: 1–4), the adjusted odds of being in a higher likelihood-of-use category were approximately 4.1 times greater, holding the other predictors constant. The environmental burden score represents participants’ perceived effects of dust storms and air pollution on asthma and should not be interpreted as a clinically validated measure of environmental exposure.

Need for additional asthma-management support was also independently associated with greater likelihood of application use (aOR = 3.17, 95% CI: 1.62–6.21, *p* < 0.001). Each one-category increase in perceived need for additional support was associated with approximately 3.2-fold higher adjusted odds of being in a higher likelihood-of-use category, holding the other predictors constant. In contrast, difficulty managing asthma without digital support (aOR = 1.45, 95% CI: 0.74–2.86, *p* = 0.282) and previous use of general health applications (aOR = 0.52, 95% CI: 0.18–1.50, *p* = 0.225) were not statistically significant. Given the modest sample size and the inclusion of four predictors, these multivariable findings should be interpreted as exploratory.

Model diagnostics indicated no problematic multicollinearity (VIF range: 1.007–1.402), and the full model provided a significantly better fit than the null model (likelihood-ratio χ^2^(4) = 51.687, *p* < 0.001). In the adjusted ordinal logistic regression model, environmental burden and perceived need for additional asthma-management support were independently associated with a greater likelihood of using the proposed application, whereas management difficulty and previous use of general health applications were not statistically significant (Table 7).

## 4. Discussion

### 4.1. Principal Findings

This study identified a substantial perceived need for and stated acceptance of a smart asthma management application among adult participants with asthma in Saudi Arabia. More than three-quarters of participants (77.9%) reported a moderate-to-high overall perceived need for such an application, 58.8% expressed moderate-to-high willingness to use it, and 66.2% indicated that they were likely or very likely to use a Saudi-based smart asthma application. These findings align with previous Saudi evidence indicating interest in mobile-supported asthma self-management despite limited previous use of asthma-specific applications. In a study of 270 Saudi adults with asthma, only 14.4% reported using mobile phones for asthma care and 4.8% had an asthma-care application, whereas 74.1% expressed a desire to use mobile phones for asthma management. The low prevalence of previous asthma-app use in the present study (4.4%), together with the reported interest in future use, may indicate a gap between expressed interest in digital asthma support and previous use of asthma-specific digital tools [40]. However, stated willingness or intention should be interpreted as attitudinal evidence rather than evidence of subsequent adoption or sustained use.

The observed interest in digital asthma management can be contextualized alongside existing literature concerning digital self-management interventions. A systematic review and meta-analysis of 24 randomized controlled trials (RCTs) involving 5155 participants reported improvements in patient-reported asthma control, including Asthma Control Test (ACT) scores (standardized mean difference (SMD) = 0.43, 95% CI: 0.35–0.51) and Asthma Control Questionnaire (ACQ) scores (SMD = −0.37, 95% CI: −0.66–−0.08), as well as several measures of asthma-related quality of life. However, significant reductions in emergency visits or hospitalizations were not demonstrated. Although these findings illustrate the broader potential of digital asthma interventions, they do not establish that the specific features preferred by participants in the present survey would yield similar clinical benefits.

Participants expressed interest in functions extending beyond conventional medication reminders, including Sehhaty integration, smartwatch connectivity, environmental alerts, emergency alerts, symptom tracking, and physician-report sharing. These preferences are consistent with broader trends in digital asthma interventions that incorporate multiple self-management functions. A large 2025 randomized clinical trial involving 901 adults reported improved asthma control following a digital asthma self-management intervention over 12 months; among participants with uncontrolled asthma at baseline, the adjusted improvement relative to usual care was 2.8 ACT points (95% CI: 2.0–3.6, *p* < 0.001). An attention-placebo-controlled trial also reported greater improvement in asthma symptom control over eight weeks with a digital self-management platform than with a limited-function version. Although these external findings provide a rationale for prospectively evaluating active symptom-monitoring and self-management functions in a future Saudi asthma application, they do not demonstrate that the particular features preferred in the present survey would independently improve clinical outcomes.

The feature preferences reported by participants also showed similarities to those previously reported by Saudi healthcare providers. Previous research involving healthcare providers identified medication reminders, information about asthma triggers, mechanisms for requesting assistance during exacerbations, and synchronization of patient information with healthcare providers and health centers as desirable application functions. This convergence suggests that these functions may warrant consideration during future user-centered application development, without implying their effectiveness or eventual uptake. Similarly, participants’ preference for Sehhaty integration indicates interest in connecting asthma-management tools with familiar national digital-health infrastructure. However, the technical feasibility, acceptability, privacy implications, actual utilization, and clinical value of such integration would require prospective evaluation.

Environmental factors were also associated with intended application use. Perceived environmental burden was positively correlated with likelihood of application use (ρ = 0.646, *p* < 0.001). In the adjusted ordinal logistic regression model, greater perceived environmental burden was associated with higher likelihood-of-use categories (adjusted odds ratio (aOR) = 4.09, 95% CI: 2.09–7.98, *p* < 0.001). The environmental burden score represents participants’ perceived effects of dust storms and air pollution on their asthma rather than objectively measured environmental exposure. Consequently, these findings indicate an association between perceived environmental burden and intended application use and should not be interpreted as evidence that environmental exposure caused asthma exacerbations or would lead to future application adoption.

Previous studies provide a broader clinical context for considering environmental information in asthma management. Ambient air pollutants such as particulate matter and ozone have been associated with asthma morbidity and exacerbations. An Eastern Province-based study reported associations between particulate matter (PM_2.5_), nitrogen dioxide (NO_2_), and carbon monoxide concentrations and pediatric asthma-related emergency department visits, whereas a Riyadh-based study reported no significant increase in pediatric acute asthma exacerbations during sandstorms. These differing findings further support a cautious interpretation of the present results, which measure perceived environmental effects rather than objectively measured exposure or environmentally attributable exacerbations.

Participants’ preference for environmental alerts suggests that such functionality may nevertheless be relevant for future application design and evaluation. Previous literature indicates that air-quality alerts and environmental education may influence preventive behavior, risk perception, and action planning, although evidence demonstrating improvements in asthma outcomes from alert systems alone remains limited. Future research could therefore evaluate whether geographically localized air-quality and dust information, when combined with individualized self-management guidance, influences exposure-avoidance behavior, symptom control, or other asthma-related outcomes. The present study cannot establish that such functionality would produce these benefits.

Perceived need for additional asthma-management support was also associated with likelihood of application use (aOR = 3.17, 95% CI: 1.62–6.21, *p* < 0.001), whereas previous general health-app use was not significantly associated with intended use (aOR = 0.52, 95% CI: 0.18–1.50, *p* = 0.225). These findings indicate an association between condition-specific support needs and stated intention to use the proposed application. Because the data are cross-sectional and the outcome represents intended rather than observed use, they cannot establish that greater support needs cause or predict subsequent application adoption. Prospective studies measuring actual uptake and sustained engagement would be required to examine this relationship.

The five proposed core features environmental alerts, medication reminders, symptom tracking, physician-report sharing, and emergency alerts—showed high internal consistency (Cronbach’s α = 0.899). This supports the reliability of their combined score within the present adult sample. However, internal consistency alone does not establish construct validity or demonstrate that these features would be effective when implemented. Further psychometric evaluation would be appropriate if this feature-needs measure is used in future research.

Overall, these findings provide formative evidence regarding perceived needs, preferences, and intended use that may inform the early-stage conceptualization and user-centered design of a Saudi-focused smart asthma management intervention. Participants expressed interest in self-management tools, environmental monitoring, emergency support, clinical information sharing, national digital-health integration, and wearable connectivity. These preferences can guide subsequent exploratory design and feasibility testing, but they should not be interpreted as evidence of actual adoption or clinical effectiveness. Future prospective studies should evaluate usability, actual uptake, sustained engagement, and clinical outcomes, followed, where appropriate, by rigorous randomized evaluation of the resulting intervention.

### 4.2. Strengths and Limitations

There are several strengths of this study. First, it provides patient-centered evidence regarding the perceived need for and intended use of a smart asthma management application among adults with asthma in Saudi Arabia. Second, it examined participants’ preferences for a broad range of application features relevant to asthma self-management and the Saudi digital-health context, including environmental alerts, symptom tracking, medication reminders, emergency support, physician-report sharing, Sehhaty integration, and smartwatch connectivity. These findings may inform future user-centered application design but should not be interpreted as evidence that specific integrations are technically feasible or clinically effective.

Third, the study assessed a broad spectrum of clinical, self-management, environmental, and digital-health-related factors potentially associated with intended application use. The five core feature-needs items demonstrated high internal consistency (Cronbach’s α = 0.899). However, internal consistency alone does not establish construct validity or one-dimensionality, and further psychometric evaluation of the questionnaire is warranted. The study also employed complementary analytical approaches, including Spearman correlation analysis to examine associations between relevant variables and intended application use, followed by multivariable ordinal logistic regression to examine factors independently associated with the likelihood of intended use after adjustment for selected covariates.

Several limitations should be considered when interpreting the findings. First, the cross-sectional design does not permit conclusions regarding causality or temporal relationships between perceived needs and intended application use. Moreover, intended use should not be interpreted as evidence of actual adoption, sustained engagement, or future clinical use. Second, the final analytical sample was modest (*n* = 68), and the multivariable regression included four predictors. The regression findings should therefore be considered exploratory and interpreted cautiously.

Third, the sample was geographically and demographically concentrated, with 94.1% of participants from the Eastern Region and 72.1% being female, which limits the generalizability of the findings to the broader population of adults with asthma in Saudi Arabia. The recruitment approach may also have introduced selection bias if individuals with greater interest in digital health or asthma self-management were more likely to participate. Fourth, the study relied on self-reported information and is therefore susceptible to recall, response, and social-desirability biases. In particular, the environmental burden measure represented participants’ perceived effects of dust storms and air pollution rather than objectively measured environmental exposure.

Finally, the preferred application features identified in this study represent participants’ perceived needs and priorities and do not establish the feasibility, usability, clinical effectiveness, or real-world uptake of these functions. Similarly, the questionnaire was exploratory, and the observed internal consistency of the core feature-needs items does not constitute comprehensive psychometric validation. Future studies should include larger and more geographically representative samples, further evaluate the questionnaire’s measurement properties, and undertake iterative co-design and prototype’s usability and feasibility testing. Subsequent prospective studies should evaluate actual uptake, sustained engagement, interoperability, clinical outcomes, patient-reported outcomes, and healthcare utilization, with controlled clinical evaluation where appropriate.

### 4.3. Implications for Smart Asthma Application Development

The present findings offer several practical considerations for the early-stage conceptualization and user-centered design of a smart asthma management application tailored to the Saudi context. Rather than being organized around a single function, a future application could incorporate complementary aspects of asthma self-management. Participants expressed substantial interest in environmental alerts, emergency support, symptom tracking, physician-report sharing, and medication reminders, while Sehhaty integration and smartwatch connectivity received particularly high perceived-need scores.

Digital asthma interventions commonly incorporate combinations of education, symptom and medication monitoring, reminders, action plans, communication with healthcare professionals, and decision-support functions [15,20]. Recent systematic-review evidence indicates that digital health interventions can improve several asthma-management outcomes, although effects vary across outcomes and the contribution of individual application components remains difficult to isolate [15]. Wearable and connected technologies are also increasingly being explored for longitudinal monitoring and personalized management of asthma and other respiratory conditions [21,22]. However, the clinical utility of such technologies depends on factors including validation, interoperability, appropriate integration into clinical workflows, and the intended clinical purpose of the collected data [21,22]. Taken together, this literature provides a rationale for prospectively evaluating a multicomponent approach, while the present findings should be interpreted as participants’ preferences rather than evidence that the proposed components would improve asthma outcomes.

The discrepancy between limited previous asthma-app use and favorable intended use in the present study is particularly relevant to the Saudi context. Only 4.4% of participants had previously used an asthma-management application, whereas 77.9% considered the proposed application moderately or highly needed, 58.8% were moderately or highly willing to use it, and 66.2% were likely or very likely to use a Saudi-based application. This pattern is consistent with an earlier Saudi study of 270 adults with asthma, in which only 14.4% reported using mobile phones for asthma care and 4.8% had an asthma-care application, despite 74.1% expressing a desire to use mobile phones for asthma management [40]. Together, these findings may indicate a gap between expressed interest in digital asthma support and previous experience with disease-specific applications. However, intended use should not be interpreted as evidence of subsequent adoption or sustained engagement.

User-centered and stakeholder-informed development may be important for translating expressed interest into an application that is usable, clinically relevant, and compatible with patients’ existing self-management practices. In an asthma-specific value-sensitive design study, Almutairi et al. identified safety, awareness, credibility, usability, accessibility, autonomy, competence, relatedness, and connectivity as stakeholder priorities and translated these values into application-design requirements [35]. Similarly, the BREATH asthma self-management education platform was developed using a user-centered design process involving adults with asthma and healthcare professionals and incorporated asthma education, asthma-control assessment, an asthma action plan, and a healthcare-provider dashboard, with formative evaluation demonstrating favorable usability [17]. Real-world qualitative evidence further suggests that digital asthma systems may support patient awareness and involvement in self-management, although sustained engagement can also depend on tailored support, clear information presentation, and appropriate integration with everyday routines and healthcare delivery [11].

The preferred functions identified in the present study also show meaningful convergence with priorities previously reported by Saudi healthcare professionals. Participants expressed interest in physician-report sharing, symptom tracking, emergency alerts, and medication reminders. In an earlier Saudi survey, healthcare professionals placed importance on medication reminders, information about asthma triggers, mechanisms for requesting assistance during asthma attacks, recording attacks and medication use, visual monitoring information such as peak-flow measurements, and linking patient data with healthcare professionals and healthcare centers [41]. This convergence suggests that a patient–provider connected model may warrant consideration during future user-centered application development rather than relying solely on a patient-facing approach. Real-world qualitative evidence further suggests that digital asthma self-management systems may support patient awareness and involvement in their care and may provide monitoring information that can support clinical follow-up [11]. However, patients and healthcare professionals may have different expectations regarding responsibility for reviewing digitally generated information. Future co-design should therefore examine what information should be shared with clinicians, how frequently it should be reviewed, which findings may require a clinical response, and how these processes could be incorporated into clinical workflows without creating excessive workload [11,26].

Environmental monitoring may be particularly relevant to the proposed application. In the present study, 69.1% of participants reported a moderate-to-high perceived impact of dust storms and 75.0% reported a moderate-to-high perceived impact of air pollution on their asthma, while 70.6% reported a moderate-to-high need for environmental alerts. Perceived environmental burden was positively correlated with likelihood of application use (ρ = 0.646, *p* < 0.001) and remained independently associated with greater likelihood of intended use in the adjusted ordinal logistic regression model (aOR = 4.09, 95% CI: 2.09–7.98, *p* < 0.001). The environmental burden score reflects participants’ perceived effects of dust storms and air pollution rather than objectively measured environmental exposure. The relevance of considering environmental information in asthma management is supported by GINA, which identifies air pollution among environmental exposures associated with asthma exacerbation risk [42]. A systematic review of interventions aimed at reducing the impact of outdoor air pollution among people with asthma identified approaches including air-quality alerts, mobile interventions, education, and policy-level strategies; however, the available evidence was limited and heterogeneous [43]. These findings suggest that environmental information and alerts may warrant consideration in the future design of a Saudi smart asthma application. However, their feasibility and clinical value should be evaluated prospectively rather than inferred from participants’ perceived needs or the observed cross-sectional associations.

The distinction between perceived environmental burden and validated individualized risk prediction is particularly important when considering dust exposure. Saudi epidemiological evidence is not uniform. For example, a pediatric study conducted in Riyadh found no significant association between daily particulate-matter concentrations or major sandstorm periods and emergency presentations, asthma severity, or hospital admissions for acute asthma [44]. Accordingly, environmental information in a future application should not be presented as a deterministic prediction that an individual patient will experience an asthma exacerbation following a particular dust event. Instead, future application development could consider providing geographically relevant environmental information, individualized education, and user-configurable alerts. The feasibility and potential effects of these functions on preventive behavior, symptom burden, asthma control, and healthcare utilization should then be evaluated prospectively.

Integration with existing digital-health infrastructure may also warrant consideration in future application development. Sehhaty integration received the highest mean perceived-need score in the present study (mean = 3.29, SD = 0.95) and was strongly positively correlated with likelihood of using the proposed application (ρ = 0.674, *p* < 0.001). This finding indicates participants’ preference for potential connectivity between a disease-specific asthma application and the broader Saudi digital-health ecosystem but should not be interpreted as evidence that integration with Sehhaty is technically, administratively, or clinically feasible. Any future integration would need to address applicable Saudi health-information governance, privacy, security, interoperability, and regulatory requirements. National interoperability specifications and health-information exchange standards would therefore need to be considered during development. Accordingly, interoperability should be treated as a technical and governance consideration requiring engagement with relevant Saudi health authorities and stakeholders rather than as an application feature whose implementation can be justified solely on the basis of participants’ stated preferences.

Interest in smartwatch connectivity was also substantial (mean perceived-need score = 3.25, SD = 1.01), and smartwatch connectivity was positively correlated with likelihood of application use (ρ = 0.590, *p* < 0.001). Wearable technologies may offer opportunities for longitudinal collection of selected physiological and behavioral information, and a randomized clinical trial of a digital asthma self-management program incorporated consumer-grade wearable integration alongside symptom logging and tailored notifications [12]. However, participants’ interest in smartwatch connectivity should not be interpreted as evidence that commercially generated measurements are clinically valid or actionable. Contemporary literature on wearable and connected technologies highlights challenges related to device accuracy and clinical validation, interoperability, privacy, digital literacy, integration into clinical workflows, cost, and equitable access [21,22,23]. Accordingly, wearable integration may warrant consideration in future application development, but its usability, feasibility, clinical relevance, and appropriate data elements should be evaluated prospectively rather than inferred from participants’ stated preferences.

Symptom tracking and physician-report sharing may help connect day-to-day self-management with professional care. GINA recommends guided asthma self-management incorporating symptom and/or peak-flow monitoring, a personalized asthma action plan, and regular clinical review [42]. A future application could therefore consider supporting structured longitudinal recording of symptoms, reliever use, medication adherence, exacerbations, and, where clinically appropriate, peak expiratory flow, with concise summaries available to patients and healthcare professionals. However, the potential usefulness and feasibility of these functions would require prospective evaluation. Such data sharing should complement scheduled or clinically indicated assessment rather than imply continuous clinical surveillance, particularly because qualitative research on digital asthma self-management has shown that patients and healthcare professionals may have different expectations regarding responsibility for reviewing digitally generated information [11].

A digital asthma action plan may therefore warrant consideration as an important clinical component of a future smart asthma application. GINA recommends that patients receive a written asthma action plan and recognizes that such plans may be provided in digital formats; the plan should include information about the patient’s usual asthma treatment, actions to take when asthma worsens, and when and how to obtain urgent medical care [42]. This provides a clinical rationale for considering the integration of symptom monitoring, emergency-support information, and individualized self-management guidance within a clinician-approved asthma action plan. However, the usability, feasibility, and clinical effectiveness of implementing such a digital action plan would require prospective evaluation.

Medication reminders may also warrant consideration, but reminder functionality alone should not be assumed to resolve medication nonadherence. Medication-taking behavior can be influenced by multiple interacting factors, and effective digital interventions should therefore address relevant behavioral determinants rather than rely solely on generic notifications. Evidence-based design work on asthma medication-adherence applications has similarly emphasized selecting behavior-change techniques according to the factors contributing to nonadherence [18]. Accordingly, medication reminders could form one component of a broader, individualized adherence-support strategy, but their effectiveness should be evaluated prospectively.

Finally, the findings may be interpreted through the lens of the Nonadoption, Abandonment, Scale-up, Spread, and Sustainability (NASSS) framework, although the questionnaire was not designed as a formal NASSS-based assessment and the framework domains were not directly measured [24]. Nevertheless, several findings can be conceptually mapped to selected NASSS domains. The condition domain is reflected in variation in participants’ perceived asthma burden and the perceived effects of environmental exposures; the technology domain is reflected in preferences for environmental alerts, monitoring functions, smartwatch connectivity, and potential Sehhaty integration; the value proposition domain is reflected in participants’ perceived need for and potential value of the proposed application; and the adopter-system domain is reflected in participants’ need for additional asthma-management support, previous digital-health experience, willingness to use the application, and intended future use. These mappings should be regarded as an interpretive framework for contextualizing the findings rather than evidence of formal assessment of NASSS domains.

Importantly, perceived environmental burden and need for additional asthma-management support remained independently associated with greater likelihood of intended application use, suggesting that prospective acceptance may be related to the interaction between participants’ perceived asthma burden, support needs, and the functionality offered by the technology. These associations should not, however, be interpreted as evidence of successful adoption or implementation. The NASSS framework emphasizes that adoption, scale-up, spread, and sustainability of health technologies are influenced by interactions among the condition, technology, value proposition, adopter system, organization, wider institutional context, and adaptation over time [24]. Organizational readiness, clinical workflows, financing, governance, regulatory requirements, and long-term adaptation were not directly assessed in the present study. Contemporary sociotechnical research similarly emphasizes that favorable patient engagement with a technology alone does not ensure its transition from initial implementation to sustained clinical practice [25]. Accordingly, these findings provide a basis for further development rather than evidence of implementation success. A future application should undergo iterative co-design and usability and feasibility testing, followed by prospective evaluation of actual uptake, sustained engagement, clinical effectiveness, implementation, and sustainability.

Beyond supporting medication adherence and symptom monitoring, a future smart asthma application could also be conceptually linked to broader digital pharmacovigilance and clinical decision-support models [45,46,47]. Structured patient-reported information on medication use, suspected adverse drug reactions, symptom deterioration, and asthma exacerbations could potentially support earlier identification of medication-related concerns and facilitate communication with healthcare professionals. Where clinically appropriate, decision-support functions could incorporate guideline-based asthma action plans, medication-use patterns, and symptom trends to provide individualized educational prompts or recommendations for seeking clinical assessment. However, patient-reported events should not be interpreted as confirmed adverse drug reactions, and automated alerts should not replace professional clinical judgment. Any pharmacovigilance or decision-support functionality would require appropriate clinical validation, data-quality safeguards, privacy protection, regulatory compliance, and integration with established healthcare reporting and clinical workflows. These capabilities represent potential directions for future development rather than functions evaluated in the present study.

## 5. Conclusions

This study indicates generally favorable perceived need for and intended acceptance of a Saudi-based smart asthma management application among adults with asthma surveyed. More than three-quarters of participants (77.9%) reported a moderate-to-high need for the proposed application, while approximately two-thirds (66.2%) indicated that they were likely or very likely to use it. Participants expressed interest in a range of integrated digital functions, including Sehhaty integration, smartwatch connectivity, environmental alerts, symptom monitoring, emergency support, medication reminders, and physician-report sharing.

Likelihood of intended application use was associated with several perceived-need, environmental, and digital-health factors. In the adjusted analysis, perceived environmental burden (aOR = 4.09, 95% CI: 2.09–7.98) and need for additional asthma-management support (aOR = 3.17, 95% CI: 1.62–6.21) were independently associated with greater likelihood of intended use, whereas previous general health-app use was not significantly associated with the outcome. These findings suggest that condition-specific needs and perceived environmental context may be important considerations in the user-centered design of a future asthma application.

Overall, the findings provide formative evidence that may inform the early-stage development of an integrated and contextually relevant smart asthma management application for Saudi Arabia. However, because this cross-sectional study assessed perceived needs and intended rather than actual application use, the findings do not establish future adoption, sustained engagement, implementation success, or clinical effectiveness. Future research should involve iterative co-design and prototype usability and feasibility testing, followed by prospective evaluation of actual uptake, sustained engagement, asthma self-management, symptom control, quality of life, healthcare utilization, and clinical effectiveness.

## Figures and Tables

**Table 1:** Demographic and Clinical Characteristics of Participants With Asthma (*n* = 68).

Variable	Category	*n*	%	95% CI
**Age (years)**	18–30	30	44.1	32.9–55.9
	31–45	18	26.5	17.4–38.0
	46–60	15	22.1	13.8–33.3
	>60	5	7.4	3.2–16.1
**Gender**	Female	49	72.1	60.4–81.3
	Male	19	27.9	18.7–39.6
**Education level**	Bachelor’s degree	43	63.2	51.4–73.7
	Secondary school	13	19.1	11.5–30.0
	Diploma	6	8.8	4.1–17.9
	Master’s degree	4	5.9	2.3–14.2
	Other	2	2.9	0.8–10.1
**Region**	Eastern Region	64	94.1	85.8–97.7
	Western Region	2	2.9	0.8–10.1
	Central Region	1	1.5	0.3–7.9
	Southern Region	1	1.5	0.3–7.9
**Duration since asthma diagnosis**	<1 year	12	17.6	10.4–28.4
	1–5 years	20	29.4	19.9–41.1
	6–10 years	13	19.1	11.5–30.0
	>10 years	23	33.8	23.7–45.7
**Frequency of asthma symptoms**	Rarely	22	32.4	22.4–44.2
	Sometimes	25	36.8	26.3–48.6
	Often	16	23.5	15.0–34.9
	Always	5	7.4	3.2–16.1
**Self-rated asthma control**	Poor	5	7.4	3.2–16.1
	Fair	30	44.1	32.9–55.9
	Good	16	23.5	15.0–34.9
	Excellent	17	25.0	16.2–36.4
**Emergency department visits due to asthma**	Never	24	35.3	25.0–47.2
	1–2 times	14	20.6	12.7–31.6
	3–5 times	18	26.5	17.4–38.0
	>5 times	12	17.6	10.4–28.4
**Hospitalizations due to asthma**	Never	48	70.6	58.9–80.1
	1–2 times	14	20.6	12.7–31.6
	3–5 times	1	1.5	0.3–7.9
	>5 times	5	7.4	3.2–16.1
**Impact of asthma on daily life**	Low	35	51.5	39.8–62.9
	Moderate	22	32.4	22.4–44.2
	High	11	16.2	9.3–26.7
**Medication adherence**	Never	16	23.5	15.0–34.9
	Sometimes	32	47.1	35.7–58.8
	Often	14	20.6	12.7–31.6
	Always	6	8.8	4.1–17.9

**Table 2:** Asthma Self-Management and Digital Health Experience Among Participants (*n* = 68).

Variable	Category	*n*	%	95% CI
**Difficulty managing asthma without digital support**	No difficulty	34	50.0	38.4–61.6
	Slight difficulty	24	35.3	25.0–47.2
	Moderate difficulty	7	10.3	5.1–19.8
	Great difficulty	3	4.4	1.5–12.2
**Self-management confidence**	Low confidence	10	14.7	8.2–25.0
	Moderate confidence	30	44.1	32.9–55.9
	High confidence	14	20.6	12.7–31.6
	Very high confidence	14	20.6	12.7–31.6
**Access to asthma education/information**	No	13	19.1	11.5–30.0
	Sometimes	31	45.6	34.3–57.3
	Yes	24	35.3	25.0–47.2
**Need for additional asthma-management support**	Not needed	18	26.5	17.4–38.0
	Slightly needed	29	42.6	31.6–54.5
	Moderately needed	8	11.8	6.1–21.5
	Highly needed	13	19.1	11.5–30.0
**Previous use of health-related mobile apps**	No	32	47.1	35.7–58.8
	Yes	36	52.9	41.2–64.3
**Previous use of an asthma-management app**	No	65	95.6	87.8–98.5
	Yes	3	4.4	1.5–12.2
**Satisfaction with health apps**	Very dissatisfied	2	2.9	0.8–10.1
	Dissatisfied	5	7.4	3.2–16.1
	Neutral	50	73.5	62.0–82.6
	Very satisfied	11	16.2	9.3–26.7

**Table 3:** Environmental Factors, Air-Quality Monitoring, and Environmental Support Needs Among Participants (*n* = 68).

Variable	Category	*n*	%	95% CI
**Effect of dust storms on asthma symptoms**	No effect	7	10.3	5.1–19.8
	Low effect	14	20.6	12.7–31.6
	Moderate effect	16	23.5	15.0–34.9
	High effect	31	45.6	34.3–57.3
**Effect of air pollution on asthma symptoms**	No effect	5	7.4	3.2–16.1
	Low effect	12	17.6	10.4–28.4
	Moderate effect	15	22.1	13.8–33.3
	High effect	36	52.9	41.2–64.3
**Air-quality monitoring**	Never	15	22.1	13.8–33.3
	Sometimes	28	41.2	30.3–53.0
	Often	15	22.1	13.8–33.3
	Always	10	14.7	8.2–25.0
**Need for environmental alerts in the app**	Not needed	9	13.2	7.1–23.3
	Slightly needed	11	16.2	9.3–26.7
	Moderately needed	16	23.5	15.0–34.9
	Highly needed	32	47.1	35.7–58.8

**Table 4:** Perceived Need for Smart Asthma Application Features Among Participants (*n* = 68).

Proposed Feature	Mean ± SD	Highly Needed, *n* (%)	Moderately or Highly Needed, *n* (%)
**Sehhaty integration**	3.29 ± 0.95	39 (57.4)	53 (77.9)
**Smartwatch connectivity**	3.25 ± 1.01	39 (57.4)	52 (76.5)
**Environmental alerts**	3.04 ± 1.08	32 (47.1)	48 (70.6)
**Emergency alerts**	2.94 ± 1.17	32 (47.1)	44 (64.7)
**Symptom tracking**	2.79 ± 1.10	24 (35.3)	41 (60.3)
**Physician-report sharing**	2.74 ± 1.14	24 (35.3)	39 (57.4)
**Medication reminders**	2.57 ± 1.25	23 (33.8)	37 (54.4)

**Table 5:** Overall Need and Acceptance of the Smart Asthma Management Application Among Participants.

Outcome	Response Category	*n*	%
Overall need	Not needed	6	8.8
	Slightly needed	9	13.2
	Moderately needed	19	27.9
	Highly needed	34	50.0
	Moderately or highly needed	53	77.9
Willingness to use	Not willing	10	14.7
	Slightly willing	18	26.5
	Moderately willing	17	25.0
	Highly willing	23	33.8
	Moderately or highly willing	40	58.8
Likelihood of using a Saudi-based app	Very unlikely	11	16.2
	Unlikely	12	17.6
	Likely	16	23.5
	Very likely	29	42.6
	Likely or very likely	45	66.2

**Table 6:** Factors Associated With Likelihood of Using a Saudi-Based Smart Asthma Management Application (*n* = 68).

Factor	Spearman ρ	95% CI	Combine with Previous Column	*p*-Value	FDR *q*-Value	*n*
**Overall perceived need**	0.709	0.545	0.844	0.0000	0.0000	68
**Environmental alerts**	0.683	0.513	0.820	0.0000	0.0000	68
**Sehhaty integration**	0.674	0.496	0.813	0.0000	0.0000	68
**Core feature-needs score**	0.667	0.511	0.789	0.0000	0.0000	68
**Emergency alerts**	0.655	0.464	0.803	0.0000	0.0000	68
**Environmental burden score**	0.646	0.471	0.781	0.0000	0.0000	68
**Symptom tracking**	0.632	0.463	0.773	0.0000	0.0000	68
**Willingness to use**	0.622	0.429	0.782	0.0000	0.0000	68
**Dust-storm impact**	0.615	0.433	0.766	0.0000	0.0000	68
**Smartwatch connectivity**	0.590	0.385	0.762	0.0000	0.0000	68
**Air-pollution impact**	0.580	0.368	0.740	0.0000	0.0000	68
**Need for additional support**	0.565	0.378	0.726	0.0000	0.0000	68
**Physician-report sharing**	0.508	0.299	0.692	0.0000	0.0000	68
**Daily-life impact of asthma**	0.402	0.186	0.595	0.0007	0.0011	68
**Medication reminders**	0.385	0.156	0.592	0.0012	0.0018	68
**Difficulty managing asthma without digital support**	0.373	0.157	0.567	0.0017	0.0025	68
**Asthma symptom frequency**	0.341	0.116	0.543	0.0045	0.0061	68
**Air-quality monitoring**	0.209	−0.049	0.458	0.0866	0.0996	68
**Access to asthma education/information**	0.097	−0.170	0.350	0.4298	0.4707	68
**Self-management confidence**	−0.021	−0.281	0.225	0.8676	0.8676	68
**Previous general health-app use**	−0.029	−0.270	0.214	0.8127	0.8496	68
**Asthma control**	−0.229	−0.473	0.022	0.0605	0.0732	68
**Previous asthma-app use**	−0.240	−0.386	−0.104	0.0483	0.0617	68

**Table 7:** Ordinal Logistic Regression Analysis of Factors Associated With Likelihood of Using a Saudi-Based Smart Asthma Management Application (*n* = 68).

Predictor	β	SE	Adjusted OR	95% CI for OR	*p*-Value
**Management difficulty**	0.373	0.346	1.45	0.74–2.86	0.282
**Previous general health-app use**	−0.658	0.543	0.52	0.18–1.50	0.225
**Environmental burden score**	**1.408**	**0.341**	**4.09**	**2.09–7.98**	**<0.001**
**Need for additional support**	**1.155**	**0.343**	**3.17**	**1.62–6.21**	**<0.001**

Note. OR = odds ratio; CI = confidence interval; SE = standard error. The dependent variable was likelihood of using a Saudi-based smart asthma management application, coded from 1 (very unlikely) to 4 (very likely). OR > 1 indicates greater odds of being in a higher likelihood-of-use category. Bold values indicate statistically significant associations (*p* < 0.05).

## Data Availability

The data presented in this study are available on reasonable request from the corresponding author.
